# Cannulation configuration and recirculation in venovenous extracorporeal membrane oxygenation

**DOI:** 10.1038/s41598-022-20690-x

**Published:** 2022-09-30

**Authors:** Louis P. Parker, Anders Svensson Marcial, Torkel B. Brismar, Lars Mikael Broman, Lisa Prahl Wittberg

**Affiliations:** 1 FLOW & BioMEx, Department of Engineering Mechanics, Royal Institute of Technology, KTH, Osquars backe 18, 100 44 Stockholm, Sweden; 2 Department of Clinical Science, Intervention and Technology at Karolinska Institute, Division of Medical Imaging and Technology, Stockholm, Sweden; 3 Department of Radiology, ECMO Centre Karolinska, Pediatric Perioperative Medicine and Intensive Care, Karolinska University Hospital and Karolinska Institute, Karolinska University Hospital, Stockholm, Sweden; 4 ECMO Centre Karolinska, Pediatric Perioperative Medicine and Intensive Care, Karolinska University Hospital, Stockholm, Sweden; 5 Department of Physiology and Pharmacology, Karolinska Institute, Stockholm, Sweden

**Keywords:** Biomedical engineering, Respiratory distress syndrome, Computational science, Fluid dynamics

## Abstract

Venovenous extracorporeal membrane oxygenation is a treatment for acute respiratory distress syndrome. Femoro-atrial cannulation means blood is drained from the inferior vena cava and returned to the superior vena cava; the opposite is termed atrio-femoral. Clinical data comparing these two methods is scarce and conflicting. Using computational fluid dynamics, we aim to compare atrio-femoral and femoro-atrial cannulation to assess the impact on recirculation fraction, under ideal conditions and several clinical scenarios. Using a patient-averaged model of the venae cavae and right atrium, commercially-available cannulae were positioned in each configuration. Additionally, occlusion of the femoro-atrial drainage cannula side-holes with/without reduced inferior vena cava inflow (0–75%) and retraction of the atrio-femoral drainage cannula were modelled. Large-eddy simulations were run for 2-6L/min circuit flow, obtaining time-averaged flow data. The model showed good agreement with clinical atrio-femoral recirculation data. Under ideal conditions, atrio-femoral yielded 13.5% higher recirculation than femoro-atrial across all circuit flow rates. Atrio-femoral right atrium flow patterns resembled normal physiology with a single large vortex. Femoro-atrial cannulation resulted in multiple vortices and increased turbulent kinetic energy at > 3L/min circuit flow. Occluding femoro-atrial drainage cannula side-holes and reducing inferior vena cava inflow increased mean recirculation by 11% and 32%, respectively. Retracting the atrio-femoral drainage cannula did not affect recirculation. These results suggest that, depending on drainage issues, either atrio-femoral or femoro-atrial cannulation may be preferrable. Rather than cannula tip proximity, the supply of available venous blood at the drainage site appears to be the strongest factor affecting recirculation.

## Introduction

Venovenous extracorporeal membrane oxygenation (VV ECMO) provides life support for refractory acute respiratory distress syndrome (ARDS) and has been under growing demand during the COVID-19 pandemic^1^. Treatment aims to increase patient oxygen saturation by draining low-oxygen blood and returning oxygen-rich blood to the venous circulation. VV ECMO becomes ineffective when a high proportion returning ECMO blood is directly drained back into the ECMO circuit, not passing to the pulmonary artery and subsequently to the arterial side.

VV ECMO can be performed with a dual lumen cannula or via one dedicated drainage and one return cannula. When using two cannulae (dual-site cannulation), the configuration is either atrio-femoral (AF), femoro-atrial (FA), or femoro-femoral. In the latter case both drainage and return occur in the same venous entity, inferior vena cava (IVC) and/or vena iliaca. In the FA configuration the drainage cannula is positioned in the IVC and the return cannula is inserted in the internal jugular vein with the tip positioned in the superior vena cava (SVC). Under AF cannulation, the flow direction is reversed, with the drainage cannula tip near the base of the SVC or in the upper right atrium (RA) and the return cannula tip positioned in the iliac vein or lower IVC. Historically, AF cannulation was routine in the United States whilst European centers favoured FA. In 1998, Rich et al. presented a study whereby a bridge was installed in the ECMO circuit allowing for the direction of flow to be switched between FA and AF. Results from 9 patients showed the FA configuration achieved a higher maximum ECMO flow rate ($${Q}_{ec})$$ (5.0 L/min vs. 4.6 L/min) and higher pulmonary arterial mixed venous oxygen saturation (89.9% vs. 83.2%) suggesting lower recirculation fraction ($${R}_{f})$$^2^. FA subsequently became the dominant configuration and remains the current recommendation from the Extracorporeal Life Support Organization (ELSO)^3,4^, though some centres still prefer AF cannulation for improved drainage and more rapid and safer conversion to jugulo-femoral veno-arterial ECMO if required. In 2020, Ling reported a retrospective comparison of patients that received FA (n = 19) and AF (n = 8) VV ECMO^5^. These results showed a higher maximum $${Q}_{ec}$$ in the AF group (4.1 L/min vs. 3.5 L/min), less positive fluid balance in the first 3 days of treatment (1.2 L vs. 3.5 L) and increased likelihood of awake ECMO in AF flow direction. These contradictory reports and varied clinical experience mean that ECMO centres still differ in their cannulation approach.

Both AF and FA cannulation are associated with unique clinical features and the treatment may need to be adjusted in response. AF is suspected to have higher ${R}_{f}$. It is thought that the proximity of the drainage site to newly oxygenated blood entering the RA may be the cause^6^. To address high ${R}_{f}$, the drainage cannula may be retracted in the SVC. In FA, sometimes the more compliant IVC collapses due to central venous hypovolemia, which may lead to “cannula chattering”^7–9^. To avoid chattering, the typical patient needs to be administered fluids, though a positive fluid balance is associated with increased in-hospital mortality^10,11^.

Through computational fluid dynamics (CFD), ECMO-induced haemodynamics can be simulated in silico. Previously, CFD has been used to assess the performance of ECMO cannulae in isolation^12^, and in the RA^13–15^, though the latter were limited to one dual lumen cannula design. The authors have previously published CFD results for a patient-averaged model of the IVC, SVC and RA under a range of vena cava flow rates to establish the dominant flow characteristics of the RA without ECMO cannulae^16^. Whilst it is difficult to quantify ${R}_{f}$ clinically (6), CFD can be applied to assess the effect of cannula repositioning and changes of ECMO/native flow rates in a controlled setting.

The primary goal of this study was to compare ${R}_{f}$ with AF and FA cannulation under ideal conditions, to examine how the unique haemodynamics of each may impact treatment effectiveness. Secondary to this, several clinical scenarios were examined: occlusion of FA drainage cannula side-holes, reduced IVC inflow to the FA cannula and retraction of the AF cannula.

## Methods

### 3D Geometries

**Figure 1:**
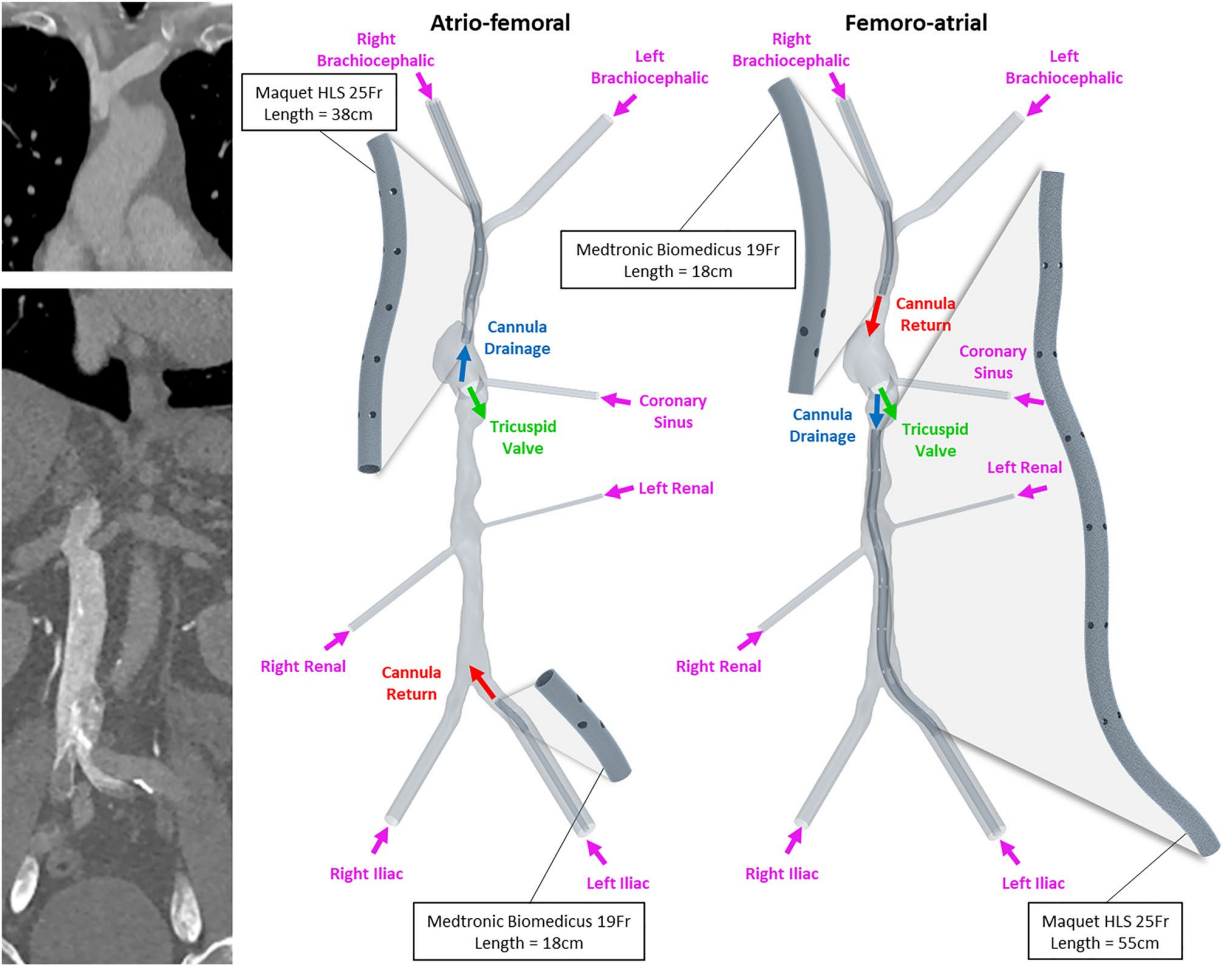
CT images showing the two separate acquisitions used to create a full reconstruction of the venae cavae and right atrium (left). The atrio-femoral and femoro-atrial cannulation models (right).

A patient-averaged CFD model of the brachial, iliac, IVC, SVC and RA, described and tested for modelling sensitivities in previous publications^16,17^, was used to simulate background venous flow. This model was constructed from two separate CT image acquisitions (Fig. 1) of four healthy subjects (3 female, 1 male, mean ± SD = 58.3 ± 3.5 years). All subjects gave informed consent. Ethics approval was obtained from the Swedish Ethical Review Authority (Ethical permit 2018/438–31) and all imaging was performed in accordance with institutional guidelines. To simulate FA and AF cannulation, geometries were reproduced for commercially available cannulae and were positioned according to an appropriate clinical application (Table 1).

**Table 1:** Cannula characteristics and positioning used to simulate atrio-femoral and femoro-atrial venovenous extra-corporeal membrane oxygenation.

Cannulation	Drainage				Return			
	product	Size (Fr)	Length (cm)*	Tip position	product	Size (Fr)	Length (cm)*	Tip position
Atrio-femoral	Maquet HLS Multistage	25	38	2 cm into RA from SVC	Medtronic Bio-Medicus	19	18	Iliac vein 2 cm prior to IVC
Femoro-atrial	Maquet HLS Multistage	25	55	IVC, 2 cm prior to the RA	Medtronic Bio-Medicus	19	18	SVC 3 cm prior to RA

*Effective length.

The cannulae were reproduced based on previously reported measurements^18^ and product data sheets using the 3D-CAD tool within the CFD solver STAR-CCM + (Version 2020.3, Siemens, Munich, Germany). To conform to the venous morphology, cannulae were constructed along vein centrelines. At several locations the diameter of the outer cannula exceeded that of the vena cava. Here, the inner wall of the cannula was defined as the new fluid boundary and any corresponding cannula side-holes were occluded, representing contact between the vein and cannula (S Fig. 1).

### Clinical scenarios

Four separate studies were conducted to assess several potential clinical scenarios.

1. *AF and FA ECMO under ideal conditions*: AF and FA ${R}_{f}$ was compared for varying ${Q}_{ec}$ (2, 3, 4, 5, 6 L/min). The linear distance between cannula tips were 290 and 111 mm, respectively.
2. *FA ECMO with occluded drainage cannula side-holes:* All side-holes covered in the drainage cannula were covered to simulate IVC collapse. All ${Q}_{ec}$ from Study 1 were simulated.
3. *FA ECMO with occluded drainage cannula side-holes and compromised IVC flow:* For all simulations in Study 2, IVC inflow was then decreased from 100%, by increments of 25%, whilst SVC inflow was constant at physiological level (2.1 L/min).
4. *AF ECMO with retraction of the drainage cannula:* to improve ${R}_{f}$ in AF ECMO, the drainage cannula may be retracted. The drainage cannula tip was by default positioned in the cross-sectional centre of the SVC and at the junction of SVC and RA for all ${Q}_{ec}$. From this position, to test the influence on ${R}_{f}$ in AF ECMO, the drainage cannula was retracted to the mid and distal SVC positions. The linear distance between cannula tips were 334 and 389 mm, respectively.

### Computational model

**Figure 2:**
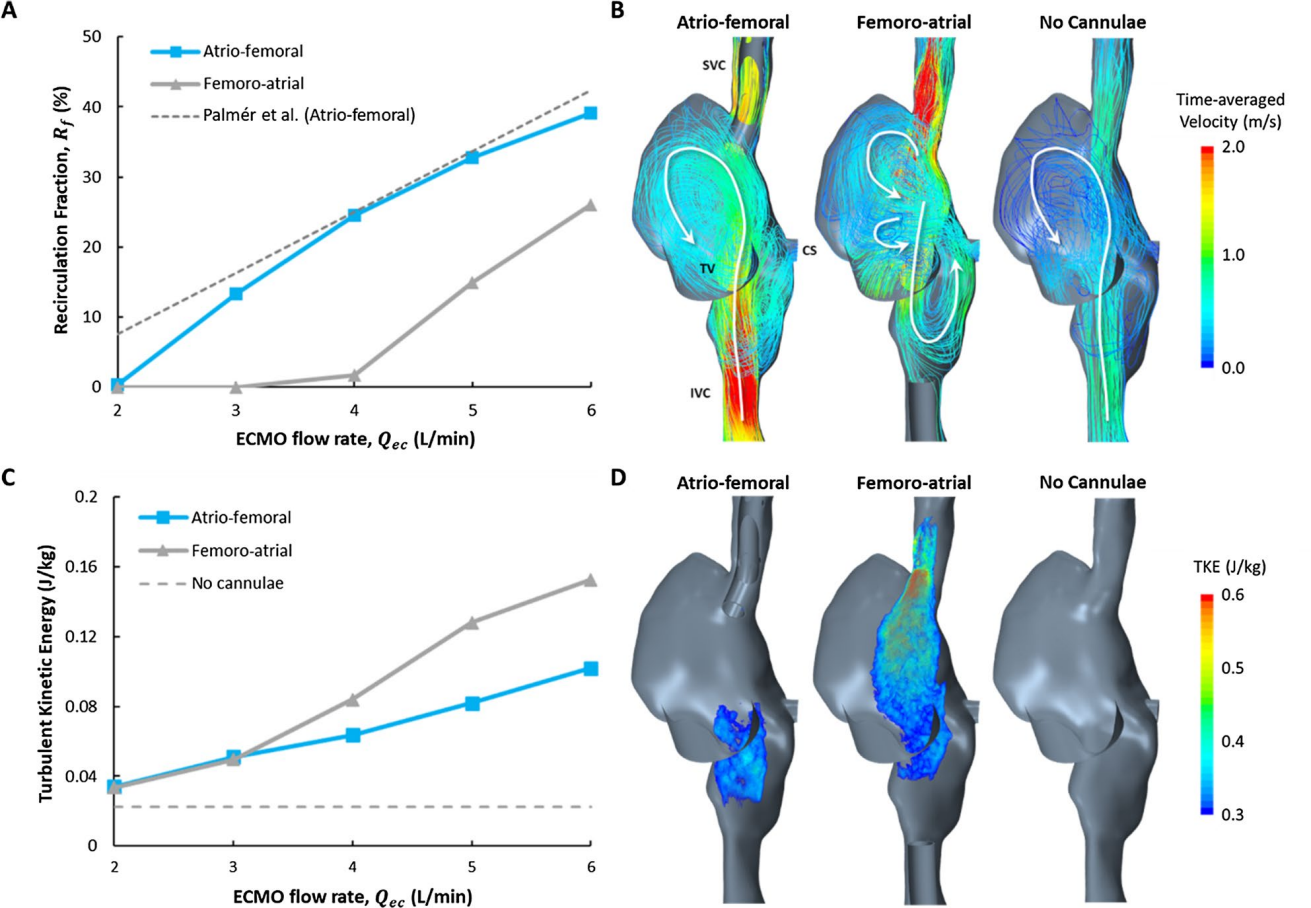
(**A**) Recirculation fraction ($${R}_{f})$$ under atrio-femoral and femoro-atrial cannulation compared to results from Palmér et al.^6^ for a range of venovenous extracorporeal membrane oxygenation (VV ECMO) flow rates. SVC = superior vena cava, IVC = inferior vena cava, TV = tricuspid valve, CS = coronary sinus. (**B**) Time-averaged velocity streamlines for both cannula configurations at 6L/min and the right atrium (RA) with no cannulae. (**C**) Volume-averaged turbulent kinetic energy (TKE) in the RA for a range of VV ECMO flow rates ($${Q}_{ec})$$. The grey dotted line represents volume-averaged RA TKE in a model without any cannulae. (**D**) A volume representation of TKE for both cannula configurations at 6 L/min and the RA with no cannulae.

The AF and FA CFD models follow the same methods as our previously published studies on RA haemodynamics^16,17^ and are outlined in the Supplemental Material (see *Computational Fluid Dynamics Model*). A grid convergence study was conducted to establish the discretisation error for velocity, ${R}_{f}$ and Turbulent Kinetic Energy (TKE) in the RA when ECMO cannula are inserted (Supplemental Material, S Fig. 2 and S Table 1).

### Clinical verification

Palmér et al. previously investigated ${R}_{f}$ during AF VV ECMO in the clinic^6^. Nine adult patients were treated with the same cannula (of varying sizes) and approximate placement as the present study. ${R}_{f}$ was then plotted as a function of ${Q}_{ec}$ based on 40 observations. This data was used to verify the ${R}_{f}$ results produced by the computational model in the present study.

### Statistics

All statistics were performed with the Real Statistics Resource Pack (Release 5.7) for Excel (Microsoft, Washington, USA). Data was tested for normality with a Shapiro–Wilk test. AF and FA ${R}_{f}$ were compared using a paired Student’s t-test.

### Ethics approval and consent to participate

All subjects gave informed consent and ethics approval was obtained from the Swedish Ethical Review Authority (Ethical permit 2018/438–31).

## Results

**Figure 3:**
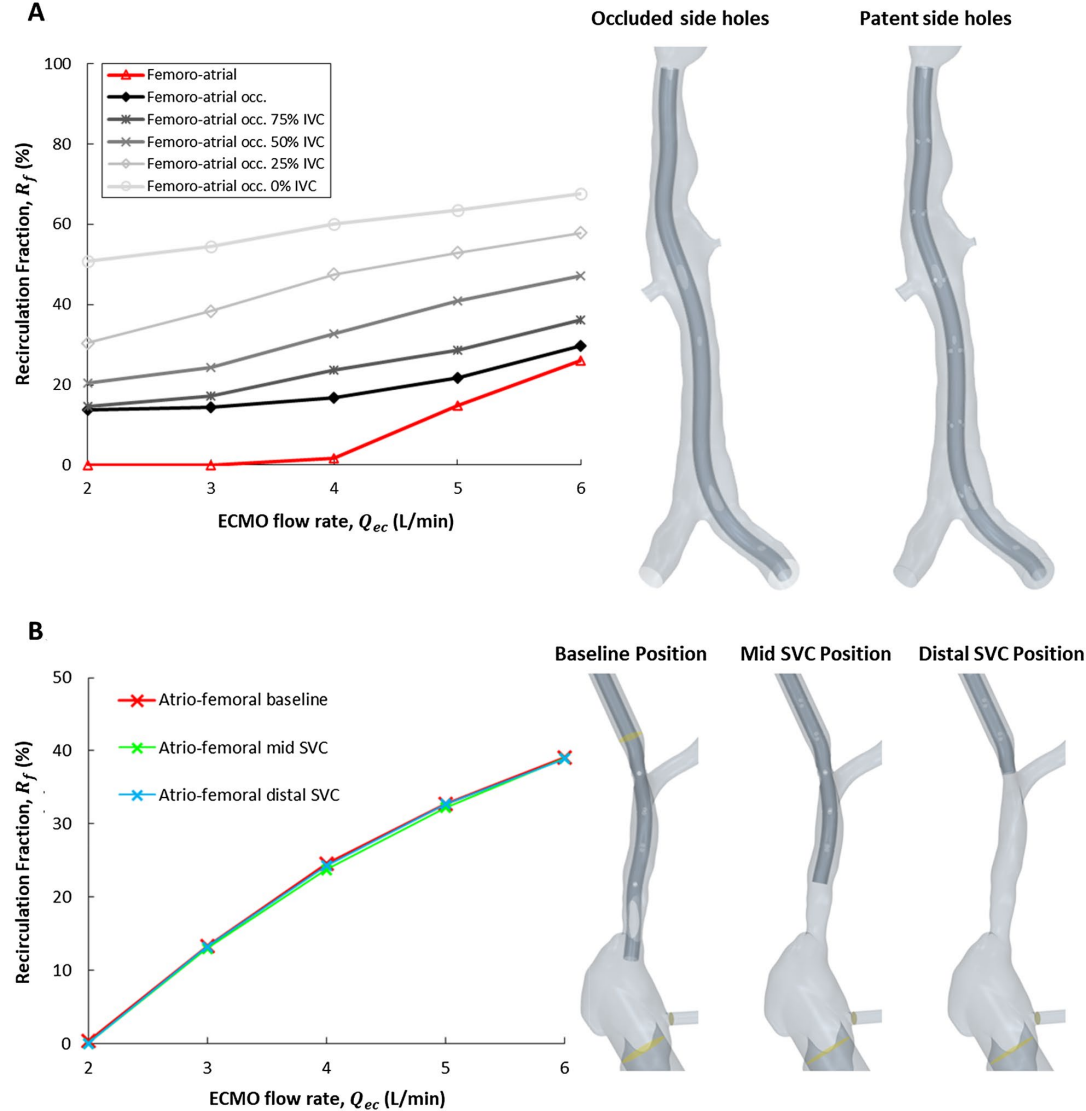
(**A**) Recirculation fraction ($${R}_{f})$$ under femoro-atrial venovenous extracorporeal membrane oxygenation (VV ECMO) with occluded and patent side-holes in the drainage cannula and for a range of decreased inferior vena cava (IVC) inflows (75–0%, 2.9–0 L/min). Occ. = occluded side-holes, SVC = superior vena cava. (**B**) $${R}_{f}$$ under atrio-femoral VV ECMO for three drainage cannula positions: baseline at junction of SVC and right atrium, mid SVC and distal SVC.

${R}_{f}$ was higher in AF than in FA for all flow rates (mean difference ± SD = 13.5 ± 7.5%, *p* = 0.011) though both have near-zero ${R}_{f}$ at 2 L/min (Fig. 2A). AF ${R}_{f}$ showed very good agreement with clinical measurements by Palmér et al. (Fig. 2A). Time-averaged velocity streamlines at 6L/min ECMO flow, in Fig. 2B show that AF cannulation creates a large rotating structure in the central RA whilst the FA cannulation model features multiple vortices of varying orientation. These structures are evident for the entire range of flow rates (Supplemental Material, S Fig. 3). Volume-averaged TKE in the RA increases with ${Q}_{ec}$ in both models. At flow rates > 3 L/min TKE under FA is increased compared to AF (mean difference ± SD = 0.04 ± 0.01 J/kg, *p* = 0.027, Fig. 2C/D).

The FA model with occluded side-holes had higher ${R}_{f}$ compared to that with patent side-holes for all ${Q}_{ec}$ (mean difference = 10.8%, *p* = 0.005, Fig. 3A). As IVC inflow was decreased from 75 to 0% in the FA model (with occluded side holes), ${R}_{f}$ increased by an average of 32% across all ${Q}_{ec}$ (Fig. 3A). There was minimal difference in AF ${R}_{f}$ between the baseline, mid SVC, and distal SVC positions (mean = 22.0%, 21.6%, 21.8%, respectively, Fig. 3B).

**Figure 4:**
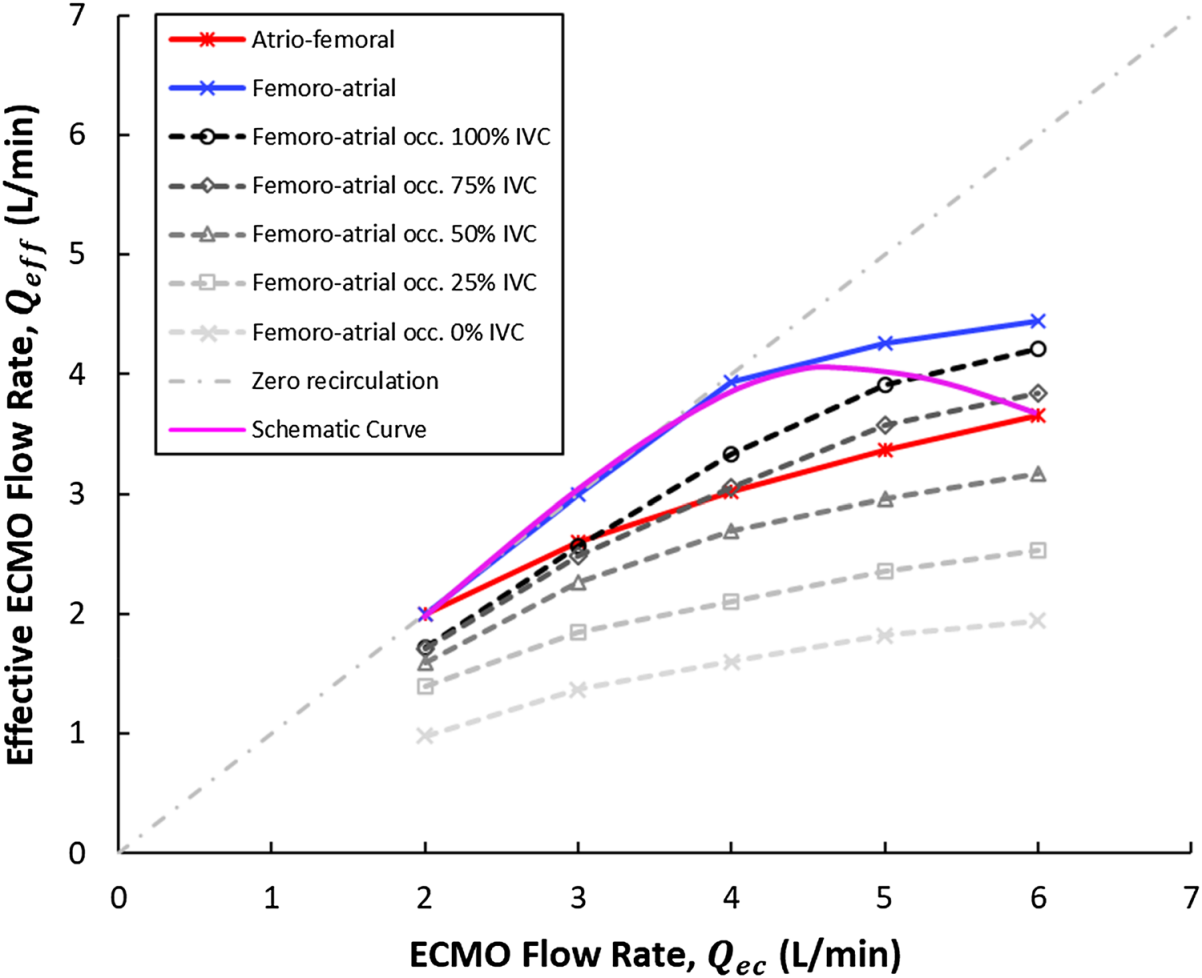
Effective extracorporeal membrane oxygenation (ECMO) flow rate ($${Q}_{eff})$$ versus ECMO flow rate ($${Q}_{ec})$$ for the atrio-femoral and femoro-atrial cannulation configurations. $${Q}_{eff}$$ obtained from the femoro-atrial model with occluded drainage cannula side-holes and reduced inferior vena cava (IVC) inflow are also plotted. Occ. = occluded side-holes. In pink we show a schematic curve of the expected relationship between $${Q}_{eff}$$ and $${Q}_{ec}$$ (circuit blood flow) proposed by Abrams et al.^19^.

Effective ECMO flow rate (ECMO flow rate—recirculation fraction) is plotted for clinical scenarios 1–3 in Fig. 4. The results show that AF or FA may be optimal depending on ECMO flow rate and clinical conditions (cannula occlusion, IVC blood supply).

## Discussion

In the present study we analysed several VV ECMO scenarios using a previously published CFD model of the SVC, IVC and RA. Initially we compared FA and AF cannulation strategies under ideal clinical conditions showing that ${R}_{f}$ increases with ${Q}_{ec}$ and is greater using AF cannulation. ${R}_{f}$ from the AF model showed good agreement with previously reported clinical data. FA ${R}_{f}$ when occluding the drainage cannula side-holes was then assessed, simulating IVC collapse. This increased ${R}_{f}$ for all flow rates relative to ideal conditions. A reduction in IVC inflow was also simulated using in the same model, resulting in further increased ${R}_{f}$. Finally, the effect of retracting the AF drainage cannula was investigated, a common adjustment made in the clinic when high ${R}_{f}$ is observed. Retraction resulted in no significant improvement with this cannula design.

### Femoro-atrial and atrio-femoral under ideal conditions

Precise relationships between ${Q}_{ec}$ and ${R}_{f}$ are unknown, owing to the practical difficulties of precise measurement in the clinic. In Study 1, FA and AF VV ECMO were examined under ideal conditions where cannula and background flows were unimpeded by morphological changes to the IVC. These results suggest that AF ${R}_{f}$ increases near-linearly from 0 to 39% as ${Q}_{ec}$ increases from 2 to 6 L/min, showing good agreement with clinical observations^6^ (Fig. 2A). FA ${R}_{f}$ is also 0% at 2L/min ${Q}_{ec}$, increasing slowly until 4L/min where ${R}_{f}$ increases more rapidly to 26% at 6L/min. A clear definition of clinically relevant ${R}_{f}$ is lacking, though Locker et al. suggest > 20%^20^. Under this threshold, and with no obstruction to cannula flow, AF ${R}_{f}$ is clinically relevant when ${Q}_{ec}$ exceeds 3L/min. For FA this threshold is 5L/min.

Another important aspect is the fluid structures induced in the RA, as captured by the CFD. The FA and AF models develop contrasting flow patterns in the RA. AF cannulation creates a single large rotating structure in the centre of the RA, a similar pattern to what the model produces with no inserted devices^16^, a pattern supported by in vivo imaging studies^21,22^. In contrast, FA cannulation generates multiple vortices, also observed in vivo though less frequently^21,22^. Under AF cannulation, total IVC inflow (ECMO and background flow) is much greater compared to SVC, reflecting the physiology of healthy adults^23^, whilst FA cannulation reverses this balance. Changes to the IVC/SVC flow rates under different ECMO configurations are likely the reason for contrasting flow patterns. The impact of such flow patterns is evident in volume-averaged RA TKE results. As ${Q}_{ec}$ is increased above 3L/min, RA TKE under FA cannulation (Fig. 2C) increases compared to the AF model as the mixing of caval flow becomes more disordered. The physiological impact of increased TKE in the RA has not been studied. However, it can be stated that high TKE is likely representative of a more turbulent combining of SVC and IVC flows, in turn requiring greater work from the atrial wall to force blood through the tricuspid valve. Additionally, turbulent blood flow is associated with endothelial cell misalignment, retraction and loss (increasing thrombosis susceptibility)^24^ and increased haemolysis^25,26^. Importantly, TKE is elevated compared to previously reported values in the RA^16^ under both cannulation methods. The clinical impact of increased RA TKE in VV ECMO should be investigated further.

### Drainage issues and femoro-atrial recirculation fraction

Some ECMO centres prefer AF cannulation to FA due to drainage issues in the IVC. It was therefore important to consider how FA ${R}_{f}$ might be affected under such circumstances. In Study 2, drainage cannula side-holes were occluded, simulating the IVC wall collapsing onto the cannula. This caused increased ${R}_{f}$ at all ${Q}_{ec}$, but the increase was most significant at low ECMO flow rates. ${R}_{f}$ at 2L/min increased from 0 to 14% after occlusion of the side-holes (Fig. 3A). With patent cannula side-holes, most of the blood is drained from the proximal holes with the cannula tip only draining a relatively small fraction of the flow. With all blood now drained through the cannula tip, the drainage site is far closer to the return cannula where blood is of a higher oxygen saturation. As well as blocking the side-holes, one would expect some reduction in the supply of blood to the IVC. We model this in Study 3 by reducing inflow to the IVC from 75 to 0%, causing increased ${R}_{f}$ (mean increase = 32%). Under the worst-case scenario, where all renal and iliac venous inflow is ceased and the ${Q}_{ec}$ is 6L/min, FA ${R}_{f}$ was 68%. Though unlikely and not compatible with survival, we included this scenario to indicate the strong influence of decreased IVC blood flow on ${R}_{f}$. Even with patent cannula side holes, reduced IVC inflow rapidly increases recirculation fraction (S Fig. 4). Studies 2 and 3 highlight the impact that practical drainage issues can have on ECMO ${R}_{f}$.

### Drainage cannula positioning and atrio-femoral recirculation fraction

When high ${R}_{f}$ is observed clinically under AF cannulation it is common to retract the SVC cannula to increase the distance between the drainage and return cannulae. Palmér et al. observed improved ${R}_{f}$ (> 5%) in only four out of ten patients when retracting their multistage AF drainage cannula (mean ± SD = 20.5 ± 3.6 mm)^6^. Importantly, the same is not observed in single stage cannulae^6^. Our simulation data confirm a minimal impact on ${R}_{f}$ when retracting a multistage cannula (Fig. 3B) and suggest a likely cause. The ${R}_{f}$ in AF cannulation appears to arise from a lack of supply of venous blood in the SVC rather than the position of the cannula tip relative to the return cannula or RA. This is clear in time-averaged velocity streamlines (Supplemental S Fig. 5). In the present study, the SVC delivers 35% of the total flow to the RA, or 2.1 L/min. When running ECMO at 2L/min the drainage cannula is supplied with all necessary venous blood and ${R}_{f}$ is near-zero. As the ${Q}_{ec}$ is increased above 2.1 L/min the extra blood must be drawn up from the RA and IVC where blood is well mixed with oxygenated return flow, increasing ${R}_{f}$ (Fig. 2A), regardless of cannula position. Conversely, under FA cannulation the total venous return in the IVC is 3.9 L/min, we accordingly see a rapid increase in ${R}_{f}$ once ${Q}_{ec}$ exceeds 4 L/min (Fig. 2A). These data suggest that rather than multistage cannula positioning being the primary factor in ${R}_{f}$, supply of venous return blood is likely more critical. Knowing how much venous blood is available to drain, whether from the SVC in AF cannulation of the IVC under FA cannulation, could help clinicians establish the ${Q}_{ec}$ at which ${R}_{f}$ is likely to become problematic.

### Clinical implications

The precise relationship between ${Q}_{ec}$ and ${R}_{f}$ has been lacking from the literature. Abrams et al. have previously presented a schematic curve depicting the relationship (Fig. 4) between ${Q}_{ec}$ and effective ECMO flow rate (${Q}_{eff}={(Q}_{ec}\times (1-{R}_{f})$)^19^ and this was included in ELSO recommendations^3^. Whilst lacking specific values, it depicts a parabolic relationship with an apex, at which point increasing ${Q}_{ec}$ would lead to a decline in ${Q}_{eff}$. Utilizing CFD in the present study, we can contribute some quantitative data to the discussion. The data from the present study in Fig. 4 supports Abrams and colleagues’ parabolic curve, however the apex occurs well beyond any reasonable ${Q}_{ec}$ for all scenarios considered in the present study. The data from this study also provides insight into the differing clinical experiences of AF and FA cannulation. Figure 4 shows that, depending on drainage conditions in the IVC, AF or FA cannulation may be preferable at any given ${Q}_{ec}$. If adequate drainage is maintained in the IVC, which may require administration of additional fluids, FA cannulation may remain the preferred option with much lower ${R}_{f}$. Although as discussed, the excessive positive fluid balance, i.e., fluid overload impacts treatment efficacy and patient outcome^10,11^. If fluid management needs to be more tightly controlled and fluid withdrawal commenced, ${R}_{f}$ may increase rapidly in FA, and AF would likely perform better based on data and clinical experience. With the emergence of dynamic extra corporeal life support^27^, whereby canulation configuration may be changed during treatment, reliable AF and FA ${Q}_{eff}$ curves become increasingly important.

### Limitations

The model relies on several simplifications, namely: rigid walls, constant inlet flow rates distributed by area and a zero-pressure outlet at the tricuspid valve. ${R}_{f}$ results for the AF model still showed good agreement with published clinical data and observations, strengthening the validity of the results despite the simplifications made. There is no clinical data on which holes might become blocked and which may be patent during chattering, we simply modelled blockage of all cannula side-holes. Whilst considering some alternative clinical scenarios, many remain. Drainage issues are more common in the IVC and so were modelled here, though such issues may arise in the SVC. For all simulations (unless IVC return flow was reduced), we applied a physiological IVC/SVC flow split of 65/35%. This is likely to differ amongst patients and may significantly impact ${Q}_{eff}$ curves.

## Conclusions

In this work, ${R}_{f}$ in VV ECMO was assessed using CFD showing good agreement with clinical observations. In all scenarios considered, the supply of venous return blood appears to be the most important factor in ECMO ${R}_{f}$ regardless of cannula positioning. Comparing AF and FA cannulation, FA showed lower ${R}_{f}$ under ideal conditions. Analysing RA flow patterns suggested that AF likely retained the normal single vortex structure whilst FA generated multiple vortices resulting in higher RA TKE at high ${Q}_{ec}$. Incorporating compromised IVC drainage in FA showed that ${R}_{f}$ may well exceed that of AF. To reposition the multistage drainage cannula in AF cannulation had no significant impact on ${R}_{f}$.

## Supplementary Information

Supplementary Information.

## Data Availability

The datasets used and/or analysed during the current study are available from the corresponding author on reasonable request.
