# Supplementary material for: Cannulation configuration and recirculation in venovenous extracorporeal membrane oxygenation
Source: Sci Rep. 2022 Sep 30;12:16379. doi: 10.1038/s41598-022-20690-x (PMC9523655; doi:10.1038/s41598-022-20690-x)
Supplement: Supplementary file 1 — Supplementary Information. [file 41598_2022_20690_MOESM1_ESM.docx]

Supplemental Material

**Cannulation Configuration and Recirculation in Venovenous Extracorporeal Membrane Oxygenation**

Louis P. Parker PhD¹, Anders Svensson Marcial PhD² ³, Torkel B. Brismar MD PhD² ³, Lars Mikael Broman MD PhD⁴ ⁵, Lisa Prahl Wittberg PhD¹.

1. FLOW & BioMEx, Department of Engineering Mechanics, Royal Institute of Technology, KTH, Stockholm, Sweden.

2. Department of Clinical Science, Intervention and Technology at Karolinska Institute, Division of Medical Imaging and Technology, Stockholm, Sweden.

3. Department of Radiology, Karolinska University Hospital and Karolinska Institutet, Stockholm, Sweden. ECMO Centre Karolinska, Pediatric Perioperative Medicine and Intensive Care, Karolinska University Hospital, Stockholm, Sweden.

4. ECMO Centre Karolinska, Pediatric Perioperative Medicine and Intensive Care, Karolinska University Hospital, Stockholm, Sweden.

5. Department of Physiology and Pharmacology, Karolinska Institutet, Stockholm, Sweden.

**Computational Fluid Dynamics Model**

A large-eddy simulation (LES) with wall-adapting local eddy-viscosity (WALE) sub-grid scale model was implemented on a polyhedral mesh (5.9-7.6M cells) with prismatic boundary layer refinement. Total venous inlet flow was 6 L/min, 65% from the IVC and 35% from the SVC [23]. These inflows were distributed by boundary area. Flow from the coronary sinus was set to 2% of the total flow [26]. The tricuspid valve was defined as a zero-pressure outlet. A non-Newtonian Quemada model [27] was implemented for blood with a haematocrit of 35% [28, 29]. Venous inflow was assumed to be constant and wall boundaries rigid. All simulations were initialized with a steady k-ω RANS simulation and then ran for 4s of physical time with velocity, wall shear stress (WSS) and turbulent kinetic energy (TKE) averaged over the final 1 s. A constant timestep of $\text{10}^{\text{-4}}$s was used with 2^nd^ order temporal discretisation. Continuity residuals converged to <$\text{10}^{\text{-5}}$. The simulations were run on the Mahti Supercomputer (CSC, Espoo, Finland) taking approximately 26,112 CPU hours each to complete.

**
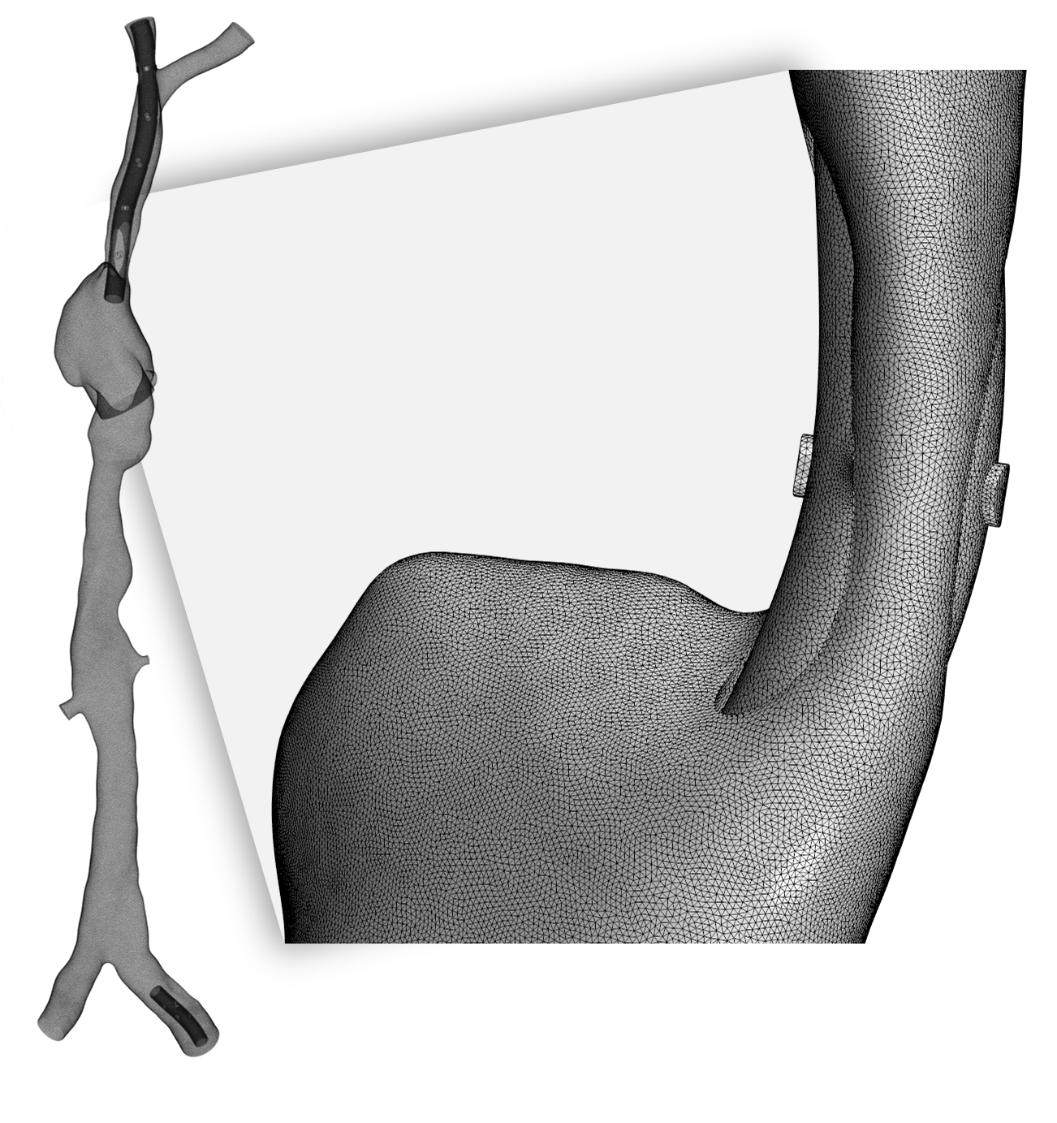
S Fig. 1.** Section of the atrio-femoral cannulation model where the drainage cannula diameter exceeded that of the inferior vena cava. Surface repair was conducted to model contact with the vena cava wall, blocking two side-holes in this example.

**S Fig.
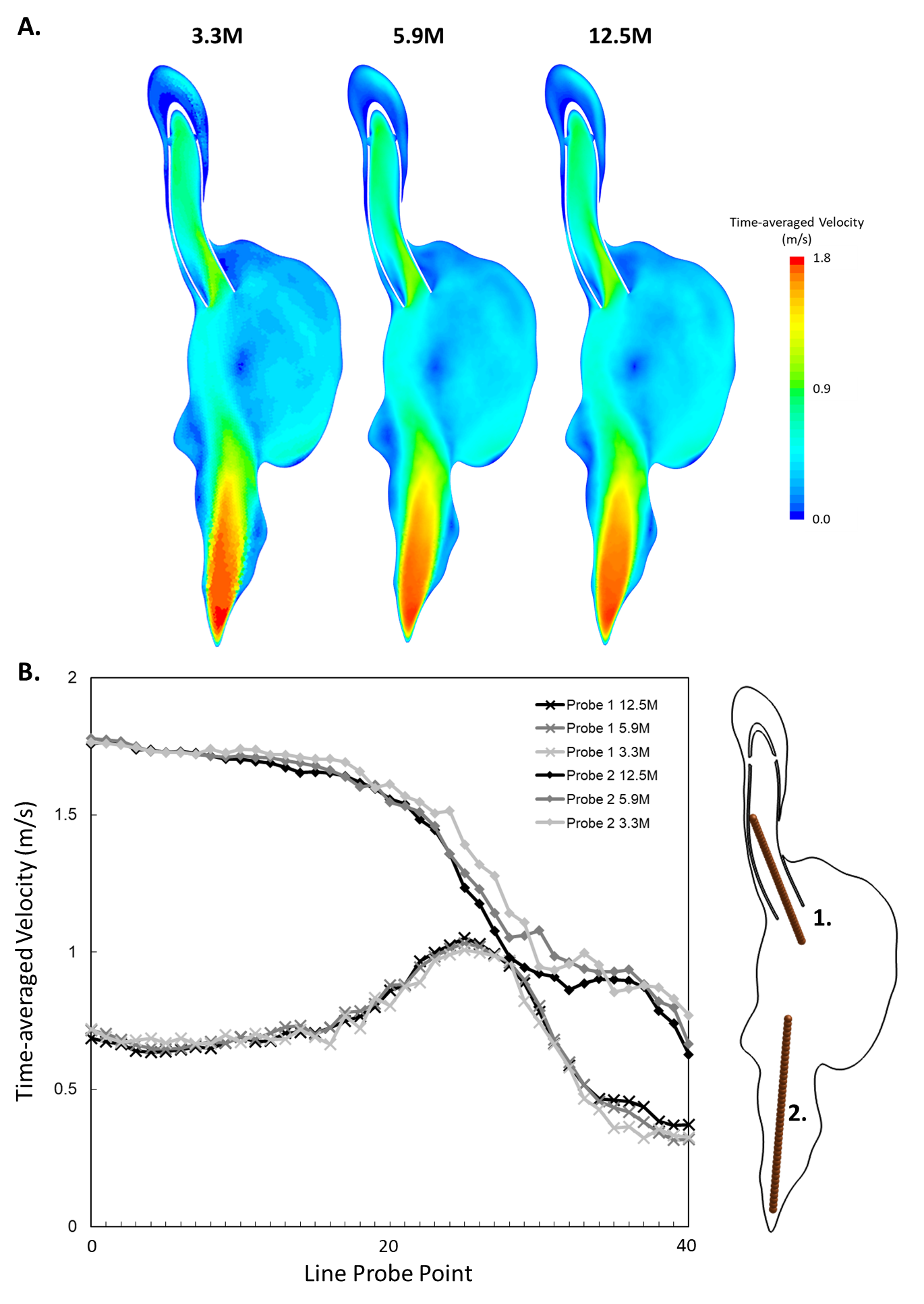
 2. A.** Time-averaged velocities on a cross-sectional plane through the three meshes used to assess grid convergence. **B.** Time-averaged velocities along two line probes placed in the right atrium, for each of the three meshes.

**
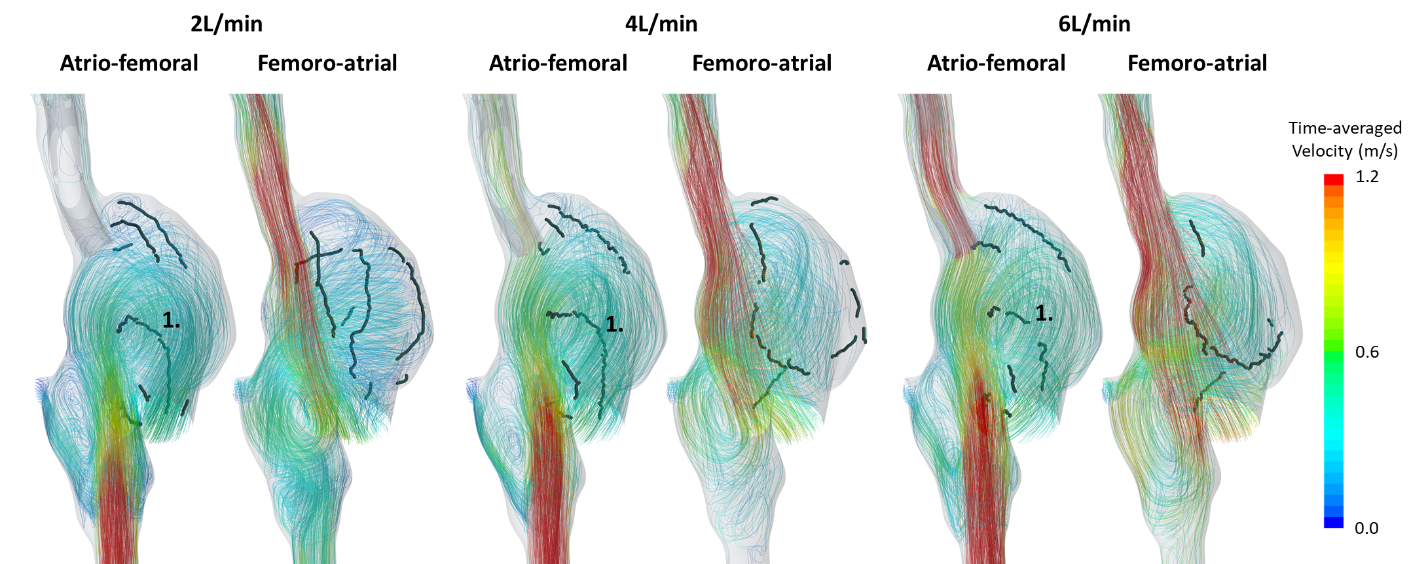
**

**S Fig. 3.** Time averaged velocity streamlines for the atrio-femoral and femoro-atrial simulations at 2, 4 and 6 L/min, vortex cores in the right atrium are indicated by black lines. AF cannulation creates a large vortex in the central atrium (1.).


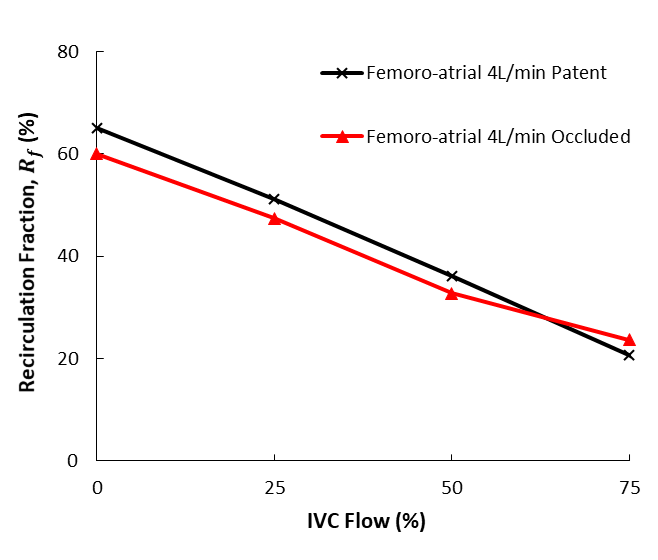


**S Fig. 4.** Comparison of patent and occluded drainage cannula side-holes with reduced IVC flow. All simulations were at an ECMO flow rate of 4L/min.


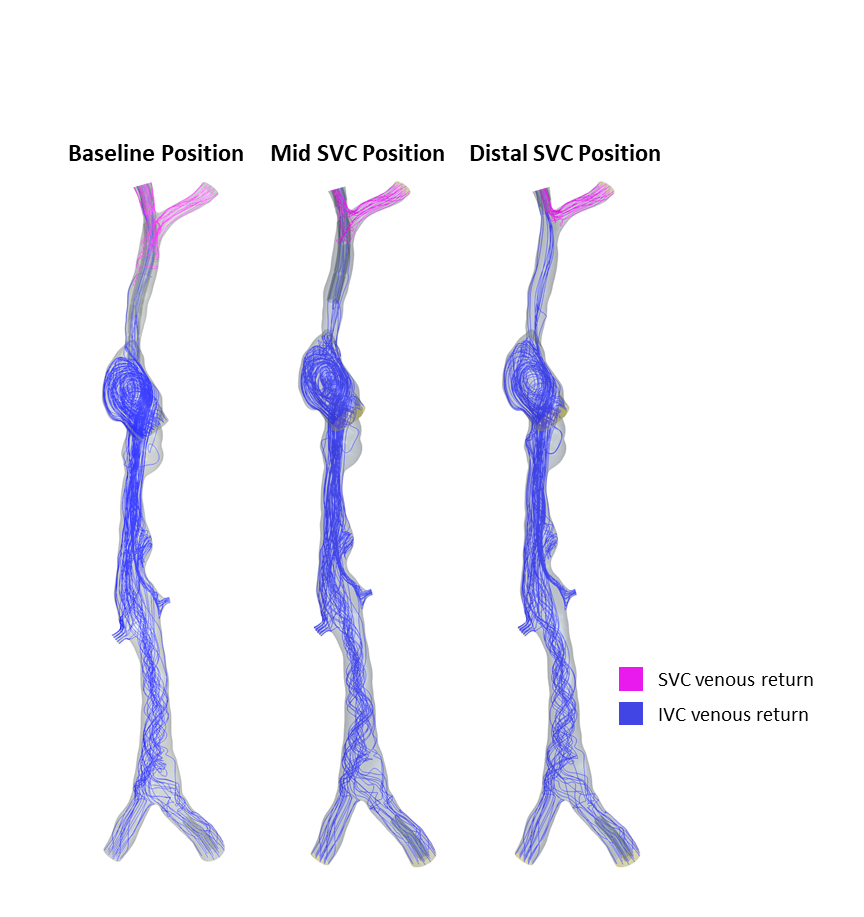


**S Fig. 5.** Time-averaged velocity streamlines for the inferior (IVC) and superior vena cava (SVC) inflows at three atrio-femoral drainage cannula positions.

| **Quantity** | **Location** | **Mean difference (3.3M vs 12.5M), %** | **Mean difference (5.9M vs 12.5M), %** |
| --- | --- | --- | --- |
| Velocity | Probe 1 | 5.84 | 2.99 |
|  | Probe 2 | 5.41 | 2.96 |
| Recirculation fraction | Drainage cannula outlet | 0.34 | 0.05 |
| Turbulent kinetic energy | Right atrium volume average | 6.03 | 5.13 |

**S Tab. 1** Grid convergence for velocity, recirculation fraction and turbulent kinetic energy. Probes 1 and 2 are shown in Figure IIB.
